# Development and validation of RNA binding protein-applied prediction model for gastric cancer

**DOI:** 10.18632/aging.202483

**Published:** 2021-02-11

**Authors:** Shuang Dai, Yan Huang, Ting Liu, Zi-Han Xu, Tao Liu, Lan Chen, Zhi-Wu Wang, Feng Luo

**Affiliations:** 1 Department of Medical Oncology, Cancer Center, West China Hospital, Sichuan University, Chengdu 610041, Sichuan Province, P.R. China; 2 Department of Medical Oncology, Lung Cancer Center, West China Hospital, Sichuan University, Chengdu 610041, Sichuan Province, P.R. China; 3 Department of Chemoradiotherapy, Tangshan People’s Hospital, Tangshan 063000, P.R. China

**Keywords:** gastric cancer, prognosis, RNA-binding proteins, bioinformatic analysis, nomogram

## Abstract

RNA-binding proteins (RBPs) have been reported to be associated with the occurrence and progression of multiple cancers, but the role in gastric adenocarcinoma remains poorly understood. The present study aims to uncover potential RBPs associated with the survival of gastric adenocarcinoma, as well as corresponding biologic properties and signaling pathways of these RBPs. RNA sequencing and clinical data of GC were obtained from The Cancer Genome Atlas (n=373) and the Gene Expression Omnibus (GSE84437, n=433) database. Tumor samples in TCGA were randomly divided into the training and internal testing group by R software. A total of 238 DERBPs were selected for univariate and multivariate Cox regression analyses. Five pivotal RBP genes (RNASE2, METTL1, ANG, YBX2 and LARP6) were screened out and were used to construct a new prognostic model. Survival relevance and prediction accuracy of model were tested via Kaplan-Meier (K-M) curves and receiver operating characteristic (ROC) curves in internal and external testing groups. Further analysis has also showed that this model could serve as an independent prognosis-related parameter. A prognostic nomogram has been eventually developed, and presents a good performance of prediction.

## INTRODUCTION

Gastric cancer (GC) is a common digestive tract tumor characterized by high incidence and mortality worldwide [1, 2]. Due to the lack of specific symptoms, most patients are diagnosed at an advanced stage, miss the optimal opportunity of surgical resection, and the outcome remains dismal [3, 4]. The Surveillance, Epidemiology, and End Results database shows that the incidence of early gastric cancer is also increasingly rising. [5]. As is known to all, even if GC patients are in the early T-stage, peritoneal metastasis is not uncommon and its prognosis is poor, especially in young people [6–8]. Over the past few decades, great advances have been made in diagnosis and treatment of GC, and a series of genes have been regarded as diagnostic or predictive biomarkers such as detection of KRAS, NRAS, ERBB2, mismatch repair (MMR) genes [9–11]. However, there are still a large number of potential biomarkers need to be further explored which can be utilized in the early diagnosis of GC, identification of micro-metastasis, detection of chemosensitivity and so on.

To examine the effective molecular regulations for cancer, although most of researchers have spent long time focusing on protein-coding gene, relatively few studies on the roles of genes coding RBPs in cancers have been found. RBPs are a kind of proteins accounting for about 7.5% of all protein-coding genes which can bind their targets with coding and non-coding RNAs to form complexes with the function of mediating fundamental biological processes including RNA processing, RNA editing, RNA production, modification, translation and so on [12, 13]. Once these proteins aberrantly alter at the expression level or function status, they may facilitate occurrence and progression of diverse diseases like carcinogenesis. Currently, multiple studies have revealed that RBPs play an essential role in cancers such as hematological malignancies, lung cancer, and gliomas by integration of “big data” and bioinformatics [14–17]. Yet, the underlying causes of the dysregulation still remain unclear [18]. To date, no studies systematically or thoroughly investigate the biological and clinical characteristics of RBPs in GC. This study attempts to investigate the relationship between RBPs and the survival, to obtain beneficial biomarkers and to develop an optimal RBP-associated prognostic model using the large-sample RNA sequencing dataset from TCGA and GEO database.

## RESULTS

### Identification of differently expressed RBP genes for GC

**Figure 1:**
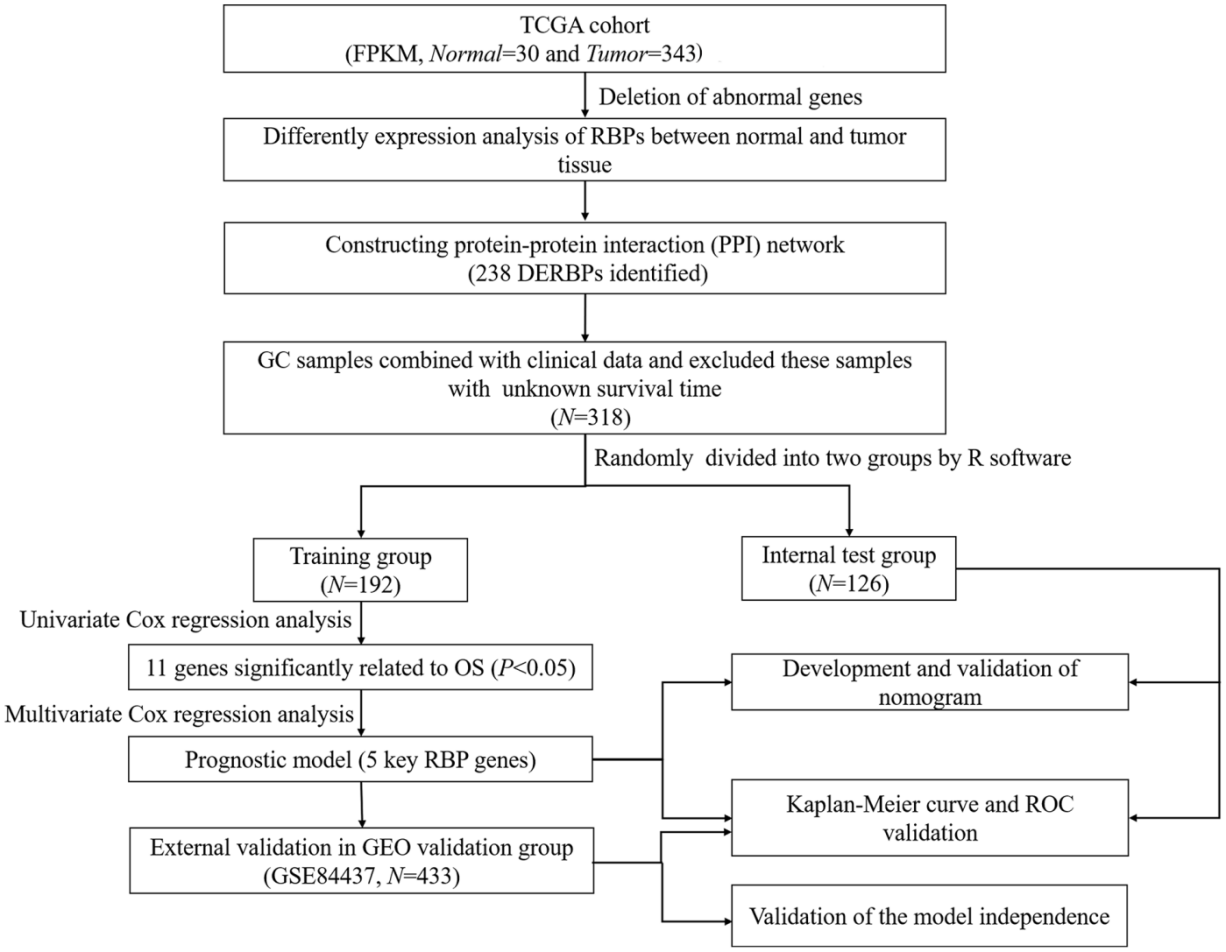
**Flow chart of constructing the five-RBP risk model.**

**Figure 2:**
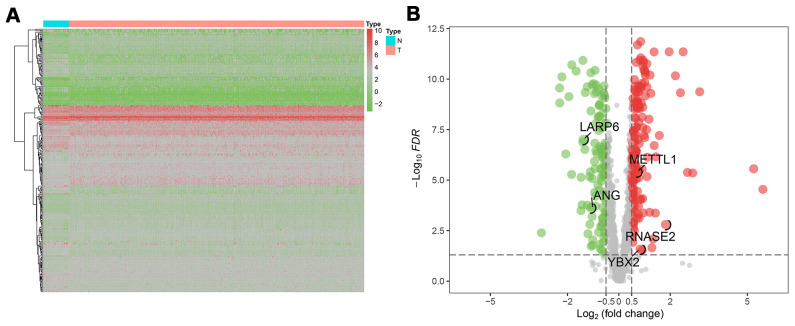
**The differentially expressed RBPs (DERBPs) in gastric cancer using The Cancer Genome Atlas (TCGA) RNA sequencing data.** (**A**) Heatmap of DERBPs; (**B**) Volcano plot. Up- and down-regulated genes are represented in red and green, respectively; FDR, false discovery rate.

The flow chart of analysis steps was listed in Figure 1 in detail. A total of 1495 RBP-associated mRNAs were identified in the TCGA dataset. At the lowest level of stringency with FDR <0.05 and |log_2_ FC| >0.5, only 272 genes (156 up-regulated and 116 down-regulated) were regarded as differentially expressed genes (DERBPs) between GC tissue and adjacent non-tumor tissue (Figure 2).

### Functional enrichment and PPI analyses of DERBPs

**Figure 3:**
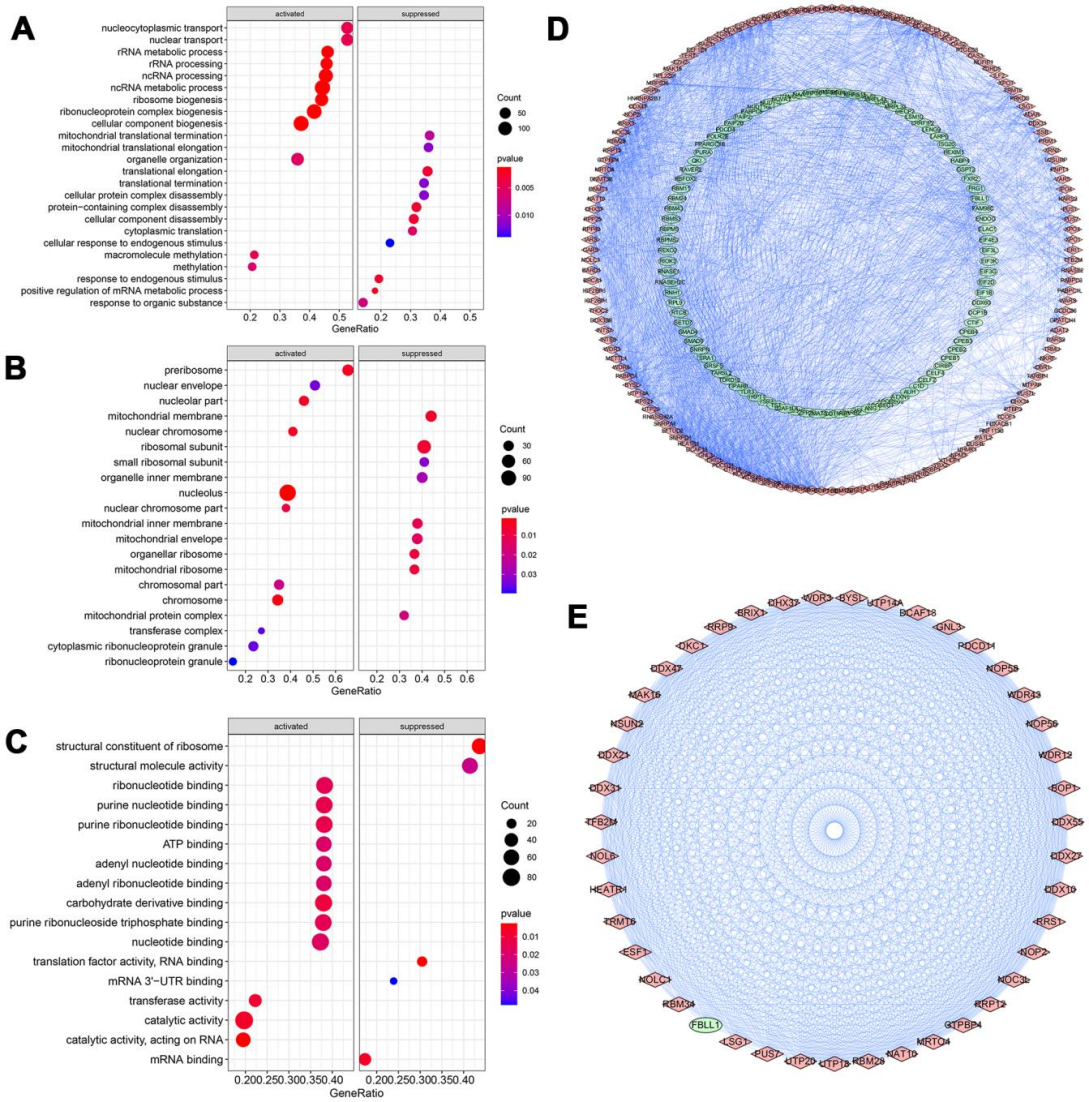
**GO Enrichment analysis and protein-protein interaction (PPI) network of DERBPs.** (**A**) Biological process; (**B**) Cellular component; (**C**) Molecular function; (**D**, **E**) PPI network of all and core module related DERBPs, respectively. Red and green nodes represent up- and down-regulated DERBPs, respectively.

All RBPs were ranked by foldchange values above, and were annotated via R package ClusterProfiler (Figure 3). The GSEA result of GO enrichment analysis indicated that RBPs in GC were apparently involved in different pathways and biofunctions (*P*-value<0.05). In biological process (BP) category (Figure 3A), RBPs activated mainly nucleocytoplasmic transport, ncRNA and rRNA processing, ribosome and ribonucleoprotein complex biogenesis, methylation, rRNA and tRNA metabolic process and gene silencing, meanwhile, RBPs significantly suppressed translational termination, protein complex disassembly, translational elongation and positive regulation of mRNA metabolic process. In the cellular component (CC) terms (Figure 3B), the main cellular components of activation were nucleolus, pre-ribosome, nuclear chromosome part, chromosome, nuclear chromosome, nuclear envelope and transferase complex. And cellular components of inhibition were mainly engaged in organellar and mitochondrial ribosome, mitochondrial membrane, inner membrane and envelope, ribosomal subunit and organelle inner membrane. In terms of the molecular function (MF) (Figure 3C), it was mainly to activate catalytic, transferase and ribonuclease activities, carbohydrate derivative binding, purine nucleotide binding, adenyl nucleotide binding, nucleotide binding, enzyme and ATP binding, methyltransferase activity and small molecule binding, while also mainly to inhibit translation factor activity of RNA binding, structural constituent of ribosome, structural molecule activity, mRNA and mRNA 3'-UTR binding. Additionally, KEGG enrichment and PPI analyses of these RBPs were also performed. KEGG analysis suggested that RBPs in GC mainly promoted ribosome biogenesis in eukaryotes and microRNAs in cancer (*P*-value<0.05). A key module which had the top score of interaction was obtained through using the MODE tool of Cytoscape software (version 3.7.2) (Figure 3D, 3E).

### Selection of prognosis-related RBPs in the training group

**Figure 4:**
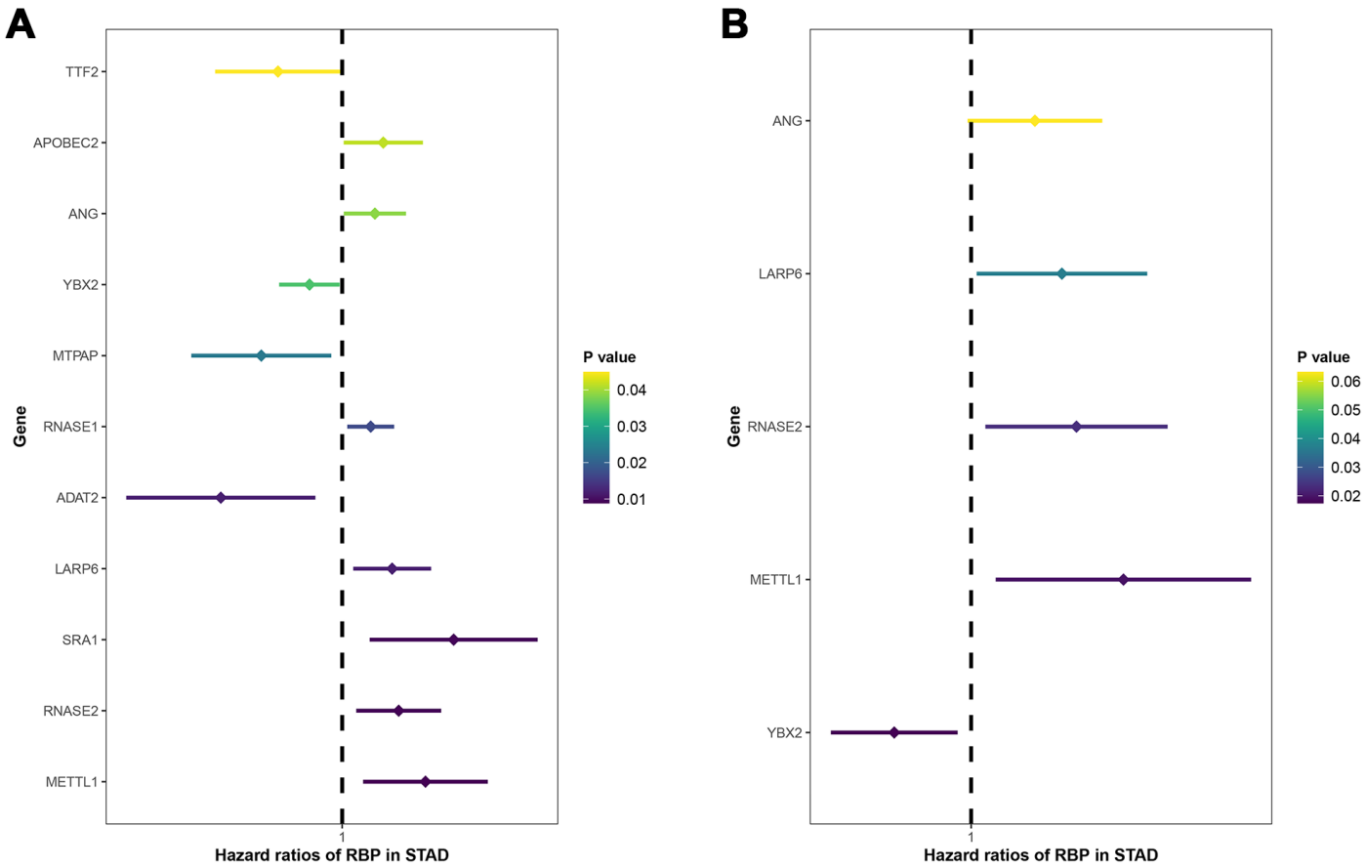
Univariate (**A**) and multivariate (**B**) Cox regression analyses for identification of key prognosis-related RBPs. X-axis: HR (95% CI); Y-axis: RBPs.

A total of 238 DERBPs screened out via PPI analysis were assessed using the univariate Cox regression analysis, and 11 survival-related genes were determined (Figure 4A). In multivariate Cox regression analysis, only these mRNAs tested through both forward and backward Cox regressions could be outputted. Five genes were finally identified, and used to construct a prognostic risk regression model (Figure 4B). Based on regression coefficients and expression levels, the total risk score was calculated: risk score = (0.3763*expression level of RNASE2) + (0.5441* expression level of METTL1) + (0.2278*expression level of ANG) + (-0.2746* expression level of YBX2) + (0.3243* expression level of LARP6). RNASE2, METTL1, LARP6 and ANG showed positive effect and probably revealed high-risk signatures. YBX2 suggested a low-risk signature. In addition, except for ANG (P=0.063), the rest of RBPs were independent prognostic factors in GC.

### External and internal validation showed the good performance of prognostic model for patients with GC

**Figure 5:**
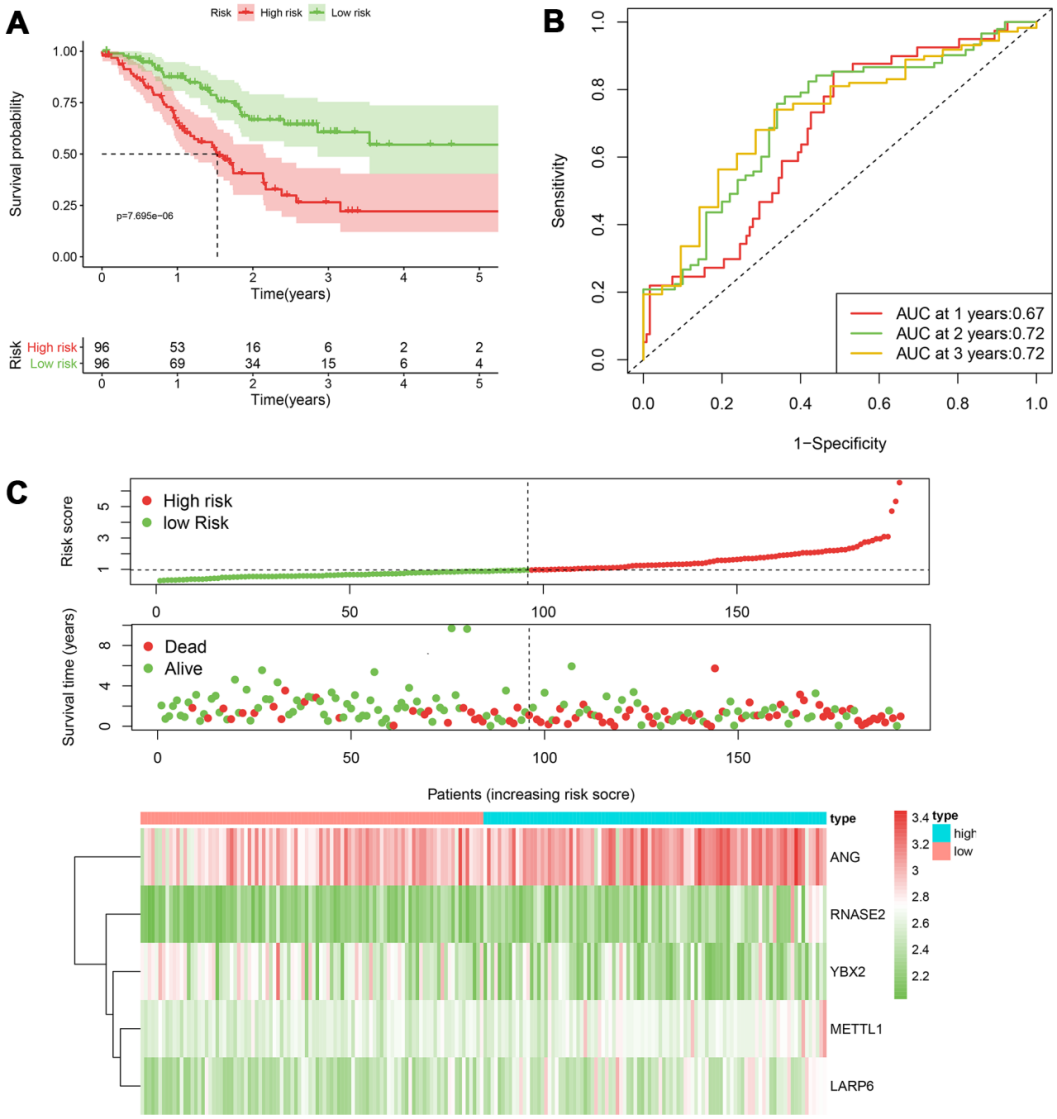
**The performance of the 5-RBP risk model in the training TCGA cohort.** (**A**) Survival curve for low- and high-risk groups; (**B**) Time-ROC curves of overall survival for validation; (**C**) Risk score distribution (upper), survival status (middle) and expression heatmap (bottom).

**Figure 6:**
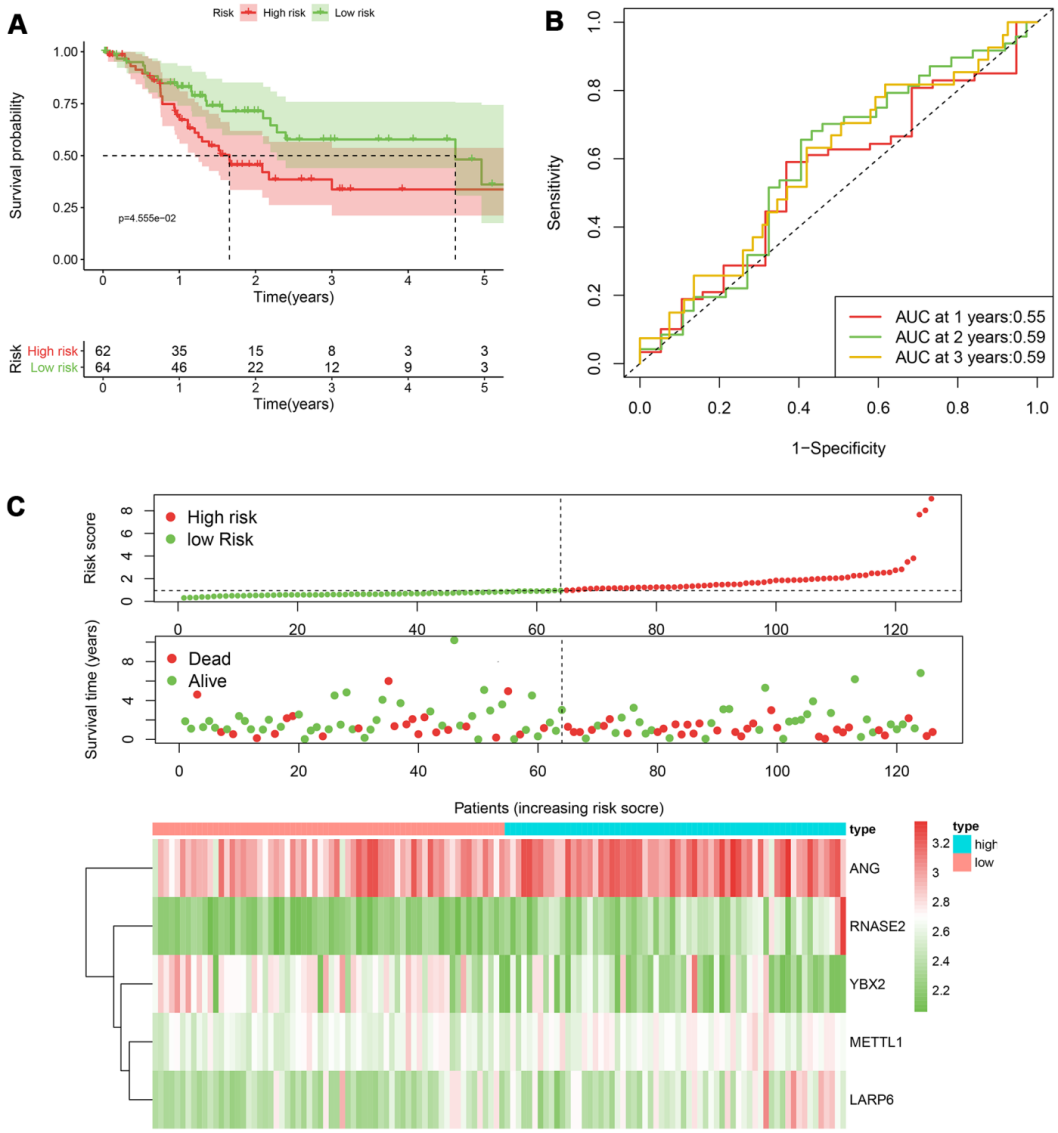
**The performance of the 5-RBP risk model in the testing TCGA cohort.** (**A**) Survival curve for low- and high-risk groups; (**B**) Time-ROC curves of overall survival for validation; (**C**) Risk score distribution (upper), survival status (middle) and expression heatmap (bottom).

**Figure 7:**
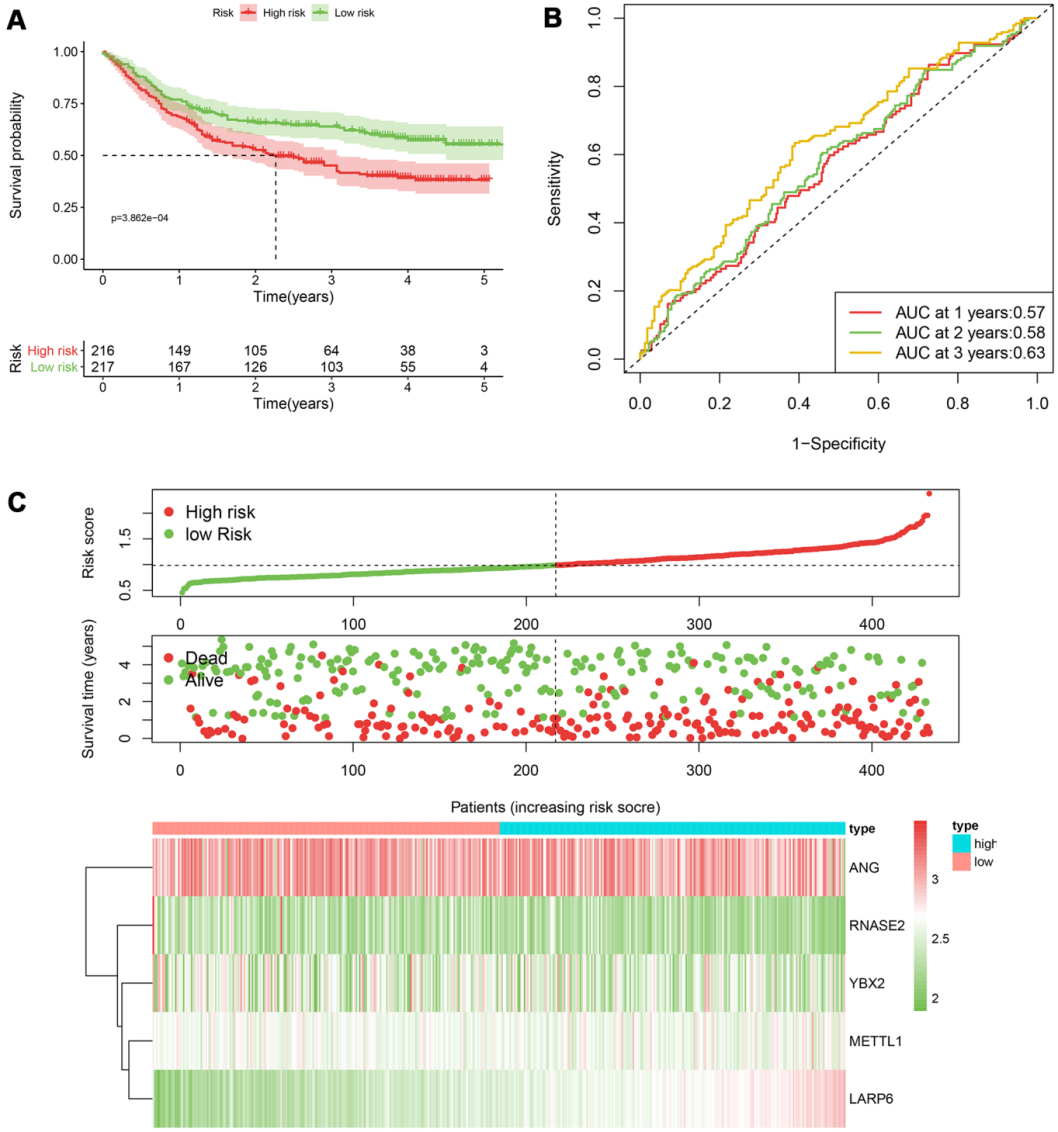
**The performance of the 5-RBP risk model in the GEO dataset.** (**A**) Survival curve for low- and high-risk groups; (**B**) Time-ROC curves of overall survival for validation; (**C**) Risk score distribution (upper), survival status (middle) and expression heatmap (bottom).

According to the median cut-off of risk score in the training cohort calculated by the prognostic formula above, patients in the training group were separated to high- and low- risk groups. Apparently prognostic differences were observed between high- and low- risk sets in the training group (*P*<0.001, Figure 5A). To verify the truth of the conclusion, the TCGA internal testing and GEO cohorts were used to validate survival significance, respectively. Similar results of survival analyses in the internal (*P*<0.001, Figure 6A) and external testing group (*P*=0.045, Figure 7A) were found. Based on the five-gene risk model, the area under the ROC curves (AUCs) for 1-, 2-, and 3-year overall survival (OS) were 0.67, 0.72, 0.72 and 0.55, 0.59, 0.59 in the TCGA training and internal validation cohort, respectively (Figure 5B, 6B). The AUCs for 1-, 2-, and 3-year OS in GEO external cohorts were 0.57, 0.58 and 0.63, respectively (Figure 7B). The heatmap, survival status of patients with GC, and risk score of the five-gene biomarker signature were also delineated in Figure 5C–7C.

### Independent prediction capacity of prognostic model

As shown in Table 1, univariate and multivariate Cox regression analyses were conducted again to evaluate the independent prediction ability of this model by comparing with other traditional clinical variables including age, gender, grade classification, tumor stage, T stage, N stage and M stage. In the TCGA entire cohort, age, tumor stage, T stage, N stage, M stage and risk score model had statistical significance in univariate analysis. Multivariate analysis further indicted that only age (*P*=0.003) and risk score model (*P*=0.043) were independent parameters related to the survival. In the GEO cohort (Table 2), age, T stage, N stage and risk score model had statistical significance in univariate analysis, while multivariate analysis showed that T stage (*P*<0.001), N stage (*P*<0.001), and risk score model (*P*<0.001) were regarded as independent prognostic factors.

**Table 1:** Univariate and multivariate Cox regression analyses of the entire TCGA cohort.

**Variables**	**Univariate analysis**			**Multivariate analysis**	
	**HR (95% CI)**	***P*-value**		**HR (95% CI)**	***P*-value**
**Age**	1.024 (1.001-1.042)	0.007		1.029 (1.010-1.049)	0.003
**Gender**	0.761 (0.527-1.098)	0.1439		——	——
**Grade**	1.184 (0.849-1.651)	0.317		——	——
**Stage**	1.550 (1.250-1.924)	<0.001		1.323 (0.862-2.030)	0.200
**T**	1.281 (1.029-1.594)	0.027		1.082 (0.795-1.473)	0.617
**M**	2.016 (1.109-3.662)	0.021		1.579 (0.700-3.563)	0.271
**N**	1.335 (1.140-1.564)	<0.001		1.172 (0.921-1.492)	0.196
**Risk Score**	1.216 (1.086-1.362)	<0.001		1.157 (1.005-1.333)	0.042

Abbreviation: HR, hazard ratio.

**Table 2:** Univariate and multivariate Cox regression analyses of the GEO cohort.

**Variables**	**Univariate analysis**			**Multivariate analysis**	
	**HR (95% CI)**	**P-value**		**HR (95% CI)**	**P-value**
**Age**	1.020 (1.007-1.032)	0.002		1.025 (1.012-1.037)	<0.001
**Sex**	0.796 (0.588-1.078)	0.141		——	——
**T**	1.740 (1.378-2.198)	<0.001		1.649 (1.290-2.110)	<0.001
**N**	1.676 (1.429-1.967)	<0.001		1.505 (1.281-1.768)	<0.001
**Risk Score**	2.436 (1.526-3.891)	<0.001		2.695 (1.621-4.481)	<0.001

Abbreviation: HR, hazard ratio.

### Expression validation of RBP genes

We downloaded the clinical immunohistochemistry specimens of GC in the Human Protein Profiles (https://www.proteinatlas.org) to observe the expression of five RBP-related biomarkers in GC. The expression of METTL1 obviously increased in tumor tissue compared to normal tissue. Besides, YBX2 tended to have a middle-strong expression in normal specimens. The expression of RNASE2 and LARP6 was slightly elevated in tumor tissue, and ANG was not collected in the Human Protein Profiles (Supplementary Figure 1).

### Construction and validation of prognostic nomogram

**Figure 8:**
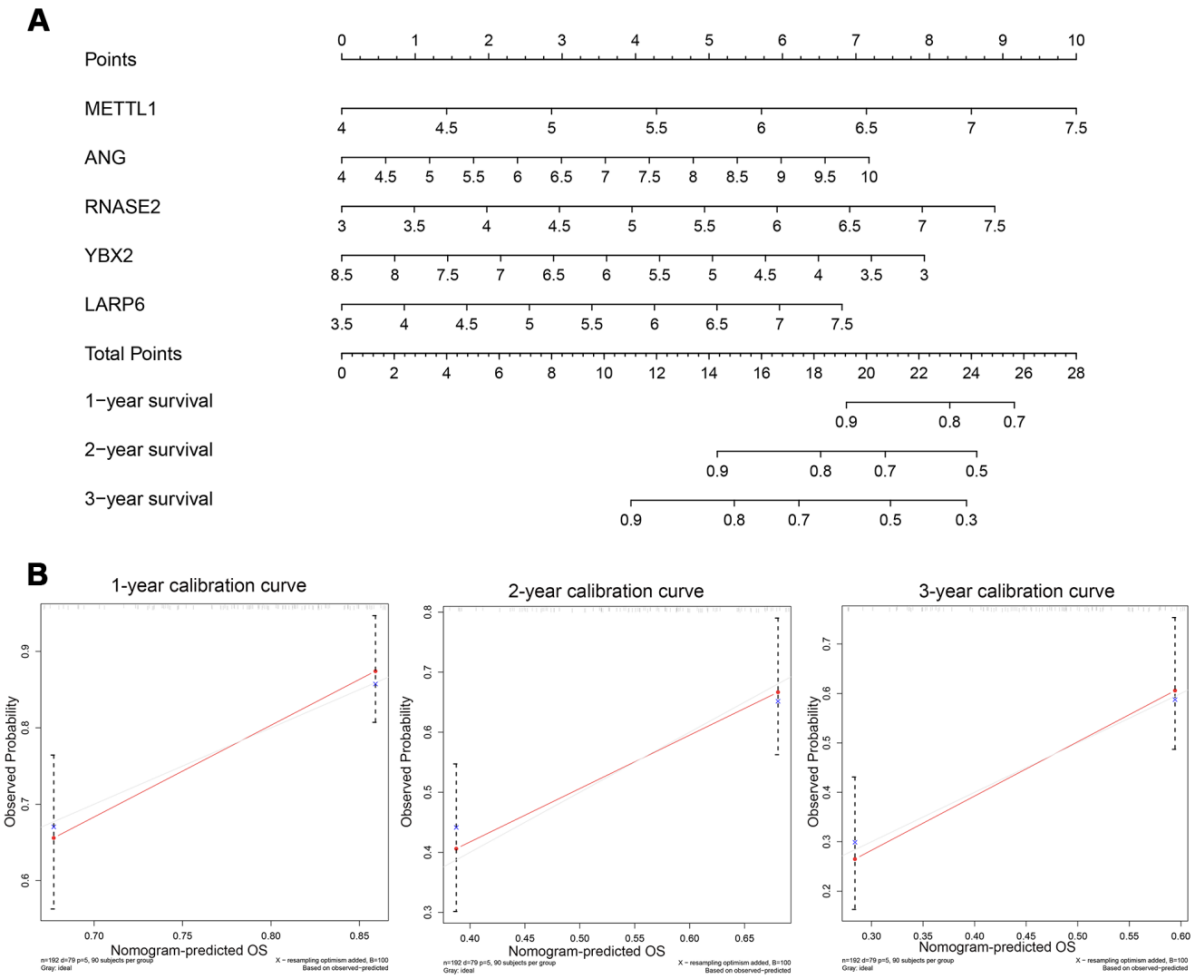
(**A**) Nomogram to predict the risk of GC patients; (**B**) Calibration curves for the prediction of 1-, 2- or 3-year overall survival.

In order to explore a prediction tool combined with five new RBP markers, we developed a nomogram to predict the survival probability of 1-, 2- and 3-year OS for GC patients (Figure 8A). We took advantage of the bootstrapping method which randomized the original set into validation set repeatedly to draw calibration curves (Figure 8B), which revealed excellent agreement between the predicted and actual survival.

## DISCUSSION

Accumulating evidence demonstrated the pivotal role of RBPs in the carcinogenesis and progression of multiple malignancies [17]. Here, a total of 238 differentially expressed RBPs were identified through strict screening. Then, we systematically analyzed the potential biological pathways and constructed PPI network based on these DERBPs. Through univariate and multivariate stepwise Cox regression analyses, we finally built a five-RBP (RNASE2, METTL1, ANG, YBX2, LARP6) predictive risk signature, and its clinical performance was further validated in the TCGA training and testing subgroup by the Kaplan-Meier and ROC methods. Moreover, GEO dataset as an independent validation group also showed similar results. Taken together, these findings may contribute to exploring novel indicators for the prognosis of GC patients.

The function enrichment analysis indicated that DERBPs were associated with the activation of nucleocytoplasmic transportation, RNA splicing, production of different RNA, RNA processing, methylation, various enzymatic activities related to metabolism, and RNA stability and modification. Meanwhile, RBPs in GC could inhibit translational termination, protein complex disassembly, translational elongation, translation factor activity and so on. Due to the critical role in stabilizing mRNAs via formation of ribonucleoprotein complexes, RBPs have been shown to be implicated in the occurrence and progression of various diseases including cancers in recent years. For examples, oncogenic RBP Lin28 confers the stemness of gastric cancer by directly binding to coding protein NRP-1 [19]. As the most prevailing modification of RNA, N6-methyladenosine (m6A) is initiated by m6A methyltransferases (METTL3, METTL14), processed by binding proteins and eliminated by demethylases (ALKBH5, FTO). All these proteins or enzymes belong to RBPs. METTL3-mediated m6A plays a pivotal role in the epithelial mesenchymal transition and metastasis of gastric cancer [20].

Also, we built a PPI network based on these DERBPs and obtained a key module consisting of 40 hub DERBPs, many of which affect tumor progression. For example, BOP1, involved in rRNA processing and gene expression, induces gastric, colorectal and liver carcinogenesis or metastasis, and correlates with the TNM staging, vascular invasion and poor disease-free survival in liver cancer [21–23]. DDX27, as RNA helicases, is critical for a series of cellular processes such as ribosome and spliceosome assembly. Therefore, it contributes to the initiation and progression of gastric and colorectal cancer, and is associated with the poor prognosis of these tumors [24, 25].

Besides, DERBPs were further identified through univariate and multivariate Cox regression analyses. Subsequently, only five DERBPs including ANG, LARP6, RNASE2, METTL1 and YBX2 were incorporated into the predictive risk model. It should be noted that METTL1 and YBX2 had been reported to be associated with other malignancies in despite of no correlation with GC. Interestingly, methyltransferase METTL1 served as a tumor suppressor and conferred chemosensitivity to cisplatin in colon cancer [26, 27]. On the other hand, Tian QH found that METTL1 facilitated cell proliferation and migration and was correlated with poor prognosis of hepatocellular carcinoma [28]. Paralleled with the Tian QH’s report, the adverse predictive RBP METTL1 seemed to play an oncogenic role in our study. As the most important Y-BOX binding protein, YBX2 binds to not only a Y-BOX element in the promoter of certain gene but also mRNAs transcribed from the parent genes [29]. Moreover, YBX2 had been reported to be associated with the initiation and progression of oral squamous cell carcinoma [29, 30].

There were inevitably several limitations in this study. Firstly, owing to the incompleteness of data from TCGA and GEO, many clinical variables cannot be enrolled in the present study. Secondly, a small number of pathological types samples with extremely poor prognosis have not been excluded. In addition, further confirmation of the existence of specific regulatory mechanism of these five RBPs, and elucidation of the clinical application require direct experimental verification and a prospective clinical trial.

## CONCLUSIONS

In conclusion, we have performed a systematic bioinformatics analysis of DERBPs, and constructed a 5-RBP prognostic model which has better performance for survival prediction in GC patients. To the best of our knowledge, this is the first report of developing a RBP related prognostic model for GC. The present study may not only provide novel insight into the role of RBPs in the tumorigenesis and progression of GC, but also develop promising diagnostic and therapeutic biomarkers for GC.

## MATERIALS AND METHODS

### Data curation and preprocessing

Gene expression information (FPKM, n=373) and corresponding survival data of patients with gastric adenocarcinoma were downloaded from TCGA database in May 2020, containing 343 tumor samples with GC and 30 adjacent non-tumor samples. Differentially expressed genes were screened followed by deletion of genes at low expression (FPKM<0.5). Furthermore, survival analysis was conducted after excluding these data with unknown survival time and survival time with 0 months (n=25). A total of 318 samples combined with the corresponding clinical data were randomly divided into training (n=192) and internal testing (n=126) groups. We also downloaded the GSE84437 cohort (n=433) as external validation group from the GEO database (https://www.ncbi.nlm.nih.gov/geo/). The study did not need the approval from the ethics committees because all data were open-access in the TCGA or GEO database.

### Functional enrichment analysis and PPI analysis

Differentially expressed genes (DEGs) with false discovery rate (FDR) cutoff of 0.05 and |log_2_(fold-change)|>0.5 were identified using the Limma package. The enrichment analyses including the GSEA analyses of Gene Ontology (GO) and Kyoto Encyclopedia of Genes and Genomes (KEGG) and protein-protein-interaction (PPI) analysis were conducted to determine the function and signaling pathways related to RBP genes. It should be noted that GSEA analysis was performed after all RBPs were ranked though the foldchange values.

### Screening of key RBP genes and establishment of prognostic model

This study carried out univariate and multivariate Cox regression analyses to identify prognostic DERBPs. Variables with *P*<0.05 detected in univariate Cox regression analysis could be enrolled in multivariate Cox regression analysis using forward and backward regression analyses to construct a prognostic model. Given the linear combination of regression coefficients with expression levels, the total risk score combined with selected key genes’ features was calculated and was used to predict the survival risk (risk score= (βgene1*expression level of gene1) + (βgene2* expression level of gene2) + (βgene3*expression level of gene3) + (βgene4* expression level of gene4) + (βgene5* expression level of gene5)). Based on the predictive formula, the risk score of each patient was calculated in the training and validation cohorts, and high- and low-risk groups were defined by the median risk score of the training set, respectively. Additionally, the predictive ability of new model was tested through the Kaplan-Meier (K-M) survival curves and time-dependent receiver operating characteristic (ROC) curves [31].

### RBP-based signature for prediction independent of clinical features

The RBP-based model together with other clinical variables including age, gender, TNM stage were subjected to the univariate Cox regression analysis. Variables associated with the survival (*P*<0.05) were then entered into the multivariate Cox regression model to determine whether the RBP-based signature was an independent prognostic factor of overall survival.

### The expression characteristics of identified RBP genes

The Human Protein Atlas database, one open-access database containing a large amount of immunohistochemical data (http://www.proteinatlas.org), was employed to analyze the expression results of the hub RBPs in GC.

### Building a predictive nomogram based on the key RBP genes

Nomogram integrating multiple variables to visualize the survival probability can provide personalized prediction of survival. The current study integrated prognosis-associated RBP genes using the TCGA training group to develop a nomogram for offering a predictive tool for patients with GC [32]. The patient can get a risk-score according to each RBP corresponding point, and then he/she can find his/her probability of the 1-, 2- and 3- overall survival. Furthermore, calibration plots (bootstrapping) were performed to evaluate the validity and accuracy of the nomogram.

### Ethics approval and consent to participate

All of data were available from open-access database. The use of data does not require additional institutional review board approval.

## Supplementary Material

Supplementary Figure 1
