# Supplementary material for: Development and validation of RNA binding protein-applied prediction model for gastric cancer
Source: Aging (Albany NY). 2021 Feb 11;13(4):5539–52. doi: 10.18632/aging.202483 (PMC7950299; doi:10.18632/aging.202483)
Supplement: Supplementary Figure 1 [file aging-13-202483-s001.pdf]

SUPPLEMENTARY FIGURE

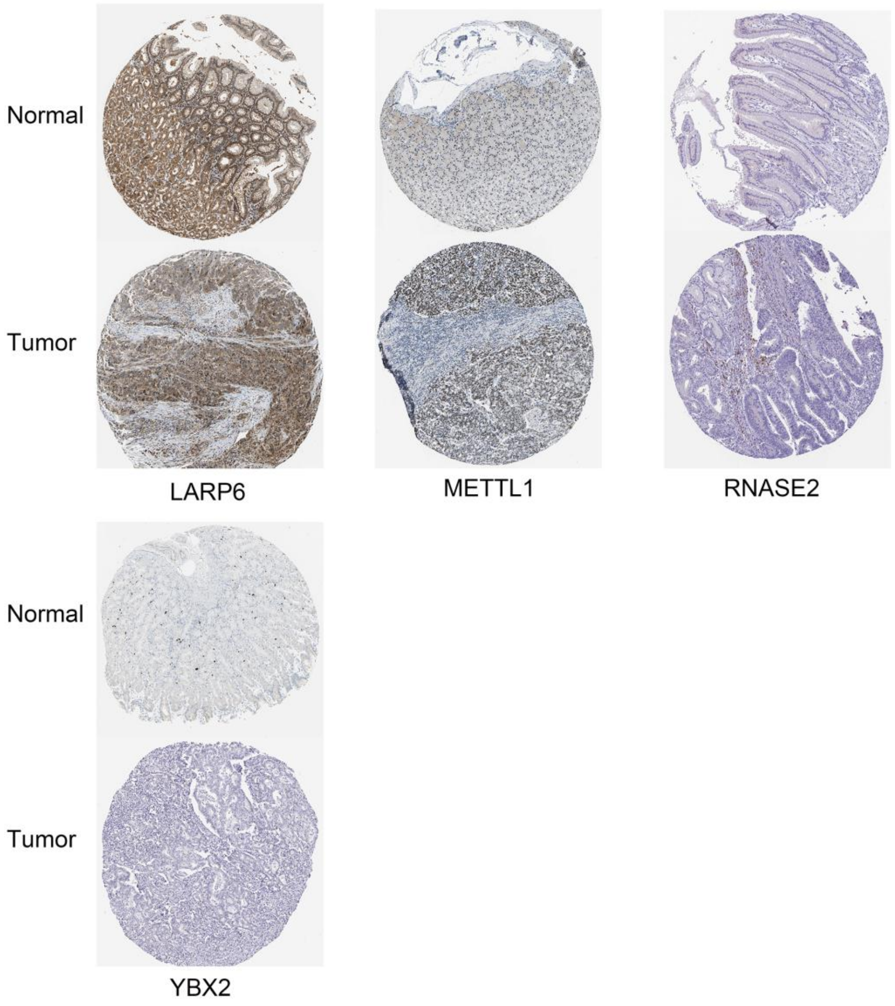

Supplementary Figure 1. Immunohistochemistry (IHC)-stained results of hub RBPs in GC and paratumor tissues from the HPA database.
